# Risk Factors for Otitis Media in Children Referred to Abuzar Hospital in Ahvaz: A Case-Control Study

**DOI:** 10.7759/cureus.9766

**Published:** 2020-08-15

**Authors:** Amir Kamal Hardani, Fatemeh Moghimi Esfandabadi, Maryam Delphi, Mohsen Ali Samir, Farzaneh Zamiri Abdollahi

**Affiliations:** 1 Department of Pediatrics, Faculty of Medicine, Abuzar Children's Hospital, Ahvaz Jundishapur University of Medical Sciences, Ahvaz, IRN; 2 Department of Audiology, Musculoskeletal Rehabilitation Research Center, Ahvaz Jundishapur University of Medical Sciences, Ahvaz, IRN; 3 Department of Audiology, School of Rehabilitation, Tehran University of Medical Sciences, Tehran, IRN

**Keywords:** otitis media, risk factor, allergic rhinitis, adenoid hypertrophy

## Abstract

Introduction

Otitis media is one of the most common causes of infection in preschool children. The most damaging complication of otitis media is temporary or permanent hearing loss. This study aimed to determine the important risk factors for otitis media.

Methods

In this case-control study, 625 children aged six months to seven years were examined from winter to spring 2020, and 53 children with otitis media were allocated to the case group and the same number to the control group. The chi-square test was used to identify the risk factors affecting otitis media, and the risk factors were compared between the case and control groups. Logistic regression was used to investigate the relationship between the incidence of otitis media and risk factors.

Results

Bivariate analysis revealed the following primary risk factors for otitis media: using pacifiers or bottle feeding, working mother, seasonal rhinitis, allergic rhinitis, tonsillopharyngitis, rhinorrhea, and adenoid hypertrophy (P<0.05). In logistic regression analysis, using pacifiers or bottle feeding (odds ratio [OR]=0.156, P=0.000), working mother (OR=0.226, P=0.000), seasonal rhinitis (OR=0.175, P=0.000), allergic rhinitis (OR=5.20, P=0.000) and adenoid hypertrophy (OR=1.57, P=0.000) were identified as the most important risk factors.

Conclusion

Adenoid hypertrophy and allergic rhinitis increased the risk of otitis media more than the other risk factors. Therefore, pediatricians should increase their awareness of the existence of these risk factors in a patient, and take the appropriate diagnostic steps and implement therapeutic care to prevent language and speech complications.

## Introduction

Otitis media, which is inflammation of the middle ear, is one of the more common childhood disorders that can lead to hearing loss. Approximately two-thirds of children of all ages are affected by otitis media [1]. Various studies have reported it having a prevalence of 15% to 40% [2,3].

According to the World Health Organization, approximately 42 million people over the age of three years suffer hearing loss due to otitis media [4]. Approximately 90% of children experience this disorder before they enter the school system. Many attacks of otitis media spontaneously resolve within three months, but in 30% to 40% of children, the disorder becomes recurrent [5,6]. Otitis media occurs in 75% of children at least three times in their first three years of life [7].

Otitis media is caused by bacteria, fungi, or viruses, and sometimes follows other infections, such as respiratory infections. One of the leading causes of otitis media is eustachian tube dysfunction. The eustachian tube may be blocked due to neoplasm, enlarged adenoids, inflammatory factors, and sinusitis, all of which cause fluid to build up in the middle ear [8].

Otitis media is seen in three forms: acute, chronic, and with effusion [9]. Antibiotics are often administered, and surgery is often performed during childhood because of otitis media. High direct and indirect costs have been estimated for otitis media, which can be a significant economic burden on health [10]. Furthermore, there are no specific guidelines in developing countries for antibiotic use for otitis media or upper respiratory tract infections. In some of these countries, the appropriate antibiotics to use are available over the counter in pharmacies [2].

The risk factors for ear infections are cultural, socioeconomic, and environmental (e.g., living in crowded places, living in large families, the duration of breastfeeding, smoking status), genetic (e.g., craniofacial anomalies such as those characteristic in Down syndrome and cleft palate), nutritional, and medical (e.g., age, gender, race, health status, history of several acute otitis episodes, rhinorrhea, allergic rhinitis, seasonal rhinitis, snoring, upper respiratory tract infections, and adenoid hypertrophy) [10-13]. No strong correlation has yet been found between otitis media and smoking, breastfeeding, or socioeconomic status [6,14]. However, some recent studies have challenged these proven factors [3,13].

Although otitis media may be asymptomatic, it can be detected during audiometric tests with temporary conductive hearing loss or by using Type B tympanometry. However, complications may exist, such as delayed language and speech, ruptured eardrum, cholesteatoma, and reading and writing impairment [15]. Other complication is mastoiditis, meningitis, acute labyrinthitis, acute petrositis. Mastoiditis is the most common intratemporal complication, and meningitis is the most commonly seen intracranial complication [16].

In developing countries, purulent infections, including chronic infections of the middle ear, mastoiditis, meningitis, cerebral abscess, infectious diseases, and epidural abscess, are essential complications of untreated middle ear infections. According to statistics, approximately 20,000 children under age five die each year from the untreated complications of otitis media [16].

Considering the above, additional consideration should be given to the rapid diagnosis and treatment of this infection and the elimination of contributing factors [17]. Therefore, due to the high prevalence of otitis media in children and its effect on hearing, speech, language, and cognitive status of children, it is necessary to study the risk factors and control them. This study aimed to investigate the factors that increase the chance of otitis media in children aged six months to seven years who were referred to Abuzar Children's Hospital by comparing the risk factors in the case and control groups.

## Materials and methods

This case-control, cross-sectional study was conducted from winter to spring of 2020 with children aged six months to seven years with otitis media who were treated at Abuzar Hospital of Ahvaz, Iran. Considering the 7% prevalence of otitis media in children, 80% power and 5% test error with a sample size of 53 children per group was calculated.

There were 625 children aged six months to seven years who were referred by pediatricians and pediatric asthma and allergy subspecialists to the Hearing Clinic of Abuzar Hospital. After receiving consent from their parents, hearing assessments began. The children first underwent otoscopy and tympanometry. Among the patients, 53 children with Type B tympanogram and healthy ear canal volume and otoscopic findings indicating the presence of otitis media (acute, chronic, and with effusion) were included in the case group. A pediatrician was consulted to confirm the diagnosis of otitis media. Then, the otitis media risk factor checklist was completed for them. Also, 53 children with Type A tympanogram normal hearing and normal otoscopy were included in the control group from among the children referred to the Abuzar Hearing Clinic. The checklist was also completed for these children.

The data collection tool was a checklist with three sections consisting of demographic information, audiology results, and otitis media risk factors. Based on previous studies, the following risk factors were extracted and studied: age, gender, working mother, education, large family, smoking, using pacifiers or bottle feeding, rhinorrhea, snoring, allergic rhinitis, seasonal rhinitis, tonsillopharyngitis, and adenoid hypertrophy [6,12].

A Reister® otoscope was used for otoscopy, and an AZ26 tympanometer was used for tympanometry. A pediatric asthma and allergy subspecialist determined the types of rhinitis and made the diagnoses of enlarged adenoids or tonsillopharyngitis. The risk factor of a large family was defined as a family with more than four members [13]. Children with impacted cerumen, Down syndrome, cleft palate, or systemic diseases such as diabetes, nephrotic syndrome, and immunodeficiency were excluded because they are susceptible to respiratory tract infections.

In addition to using descriptive statistics of the mean and standard deviation, in the data analysis, the chi-square test was used to examine the bivariate association between each of the 13 risk factors and otitis media and to compare the case and control groups. Multivariate logistic regression analysis was also used for variables obtained at a significance level of less than 0.05 in the bivariate analysis. The tests were considered significant at 0.05. The data were analyzed using IBM SPSS Statistics for Windows, Version 22.0 (IBM Corp., Armonk, NY).

## Results

Table 1 separately shows the demographic characteristics of the two groups. The mean age of the subjects in the case and control groups was 40.52±20.39 and 36.03±18.12 months, respectively, with no significant difference between them (P=0.609). As Table 1 shows, there was no significant difference between the two age groups in terms of age, sex, or education. However, the percentage of working mothers was higher in the case group (P=0.011).

**Table 1:** Distribution of demographic characteristics in the two groups

Variable		Case		Control		P-value
		Number	Percent	Number	Percent	
Age	>2 years	18	34	16	30.0	0.91
	2-3	15	28.3	17	32.1	0.91
	3-4	8	15.1	11	2.8	0.62
	4-5	6	11.3	4	7.5	0.79
	>5	6	11.3	5	9.4	0.83
Sex	Female	28	52.8	26	49.1	0.65
	Male	25	47.2	27	50.9	
Education	Literate	46	86.8	45	84.9	0.780
	Illiterate	7	13.2	8	15.1	
Occupation	Employed	29	54.7	16	30.2	0.011
	Unemployed	24	45/3	37	69/8	

Table 2 shows the distribution of risk factors in the case and control groups. Risk factors with a significance level of less than 0.05 in the bivariate analysis were included in the logistic regression analysis. These variables included: using pacifiers or bottle feeding, working mother, seasonal rhinitis, allergic rhinitis, tonsillopharyngitis, adenoid hypertrophy, and rhinorrhea.

**Table 2:** Distribution of otitis media risk factors in the two groups

Risk Factor	Case		Control		P-value
	Number	Percent	Number	Percent	
Use of a pacifier or bottle	39	73/6	20	37/7	0.000
Smoking	24	45/3	28	52/8	0.437
Seasonal rhinitis	40	75/4	16	30/2	0.000
Allergic rhinitis	37	71/7	24	45/3	0.011
Tonsillopharyngitis	25	47/2	12	22/6	0.008
Adenoid hypertrophy	38	71/1	15	28/3	0.000
Vaccination	50	94/3	52	98/1	0.308
Large family	32	60/4	28	52/8	0.433
Persistent rhinorrhea	30	56/6	8	15/1	0.006
Low weight	23	53/4	18	34	0.319
Snoring	22	41/5	15	28/3	0.154

Of the children with otitis media, 73.6% had a history of using pacifiers or bottle feeding, which was significantly different from the control group (P=0.000). As Table 2 shows, 75.4% of children with otitis had seasonal rhinitis, 71.7% had allergic rhinitis, 47.2% had tonsillopharyngitis, 71.7% had adenoid hypertrophy, and 56.6% had rhinorrhea, and these were significantly different from those of the control group. No correlation was observed between the incidence of otitis media and other risk factors (P>0.05).

Logistic regression analysis was performed to investigate the simultaneous effect of the mentioned variables on the incidence of otitis media. According to the logistics model, with 50% confidence, the risk of developing otitis media was predictable by using five risk factors of pacifiers or bottle feeding, seasonal rhinitis, allergic rhinitis, adenoid hypertrophy, and working mother. The results of logistic regression showed that using pacifiers or bottle feeding, seasonal rhinitis, allergic rhinitis, adenoid hypertrophy, and working mothers are significant risk factors for otitis media.

According to Table 3, the first risk factor that has the greatest impact on the incidence of otitis media is allergic rhinitis, such that children with allergic rhinitis are 5.20 times more likely to develop otitis media. Also, children with adenoid hypertrophy are 1.15 times more likely to develop otitis media. Risk factors of seasonal rhinitis, using pacifiers, and working mothers were also significant in the logistics model, indicating the impact of these factors on the incidence of otitis media.

**Table 3:** Logistic regression analysis results in examining otitis media risk factors

Risk Factor	Odds Ratio	95% Confidence Interval	P-value
Allergic rhinitis	5.20	(1.06-25.46)	0.000
Adenoid hypertrophy	1.157	(0.04-4/58)	0.000
Use of a pacifier or bottle	0.156	(0.05-0.58)	0.000
Seasonal rhinitis	0.175	(0.04-0.65)	0.000
Occupation	0.226	(0.07-0.67)	0.000

## Discussion

In the present study, we found no significant relationship between the incidence of otitis media and demographic characteristics such as age, gender, and educational level. This was consistent with a cohort study by Telee et al., but contrary to the findings of a study by Sophia et al. that emphasized the role of gender and age in the incidence of otitis media [5,6].

The results of the present study showed that allergic rhinitis has a significant relationship with the incidence of otitis media such that allergic rhinitis increases the risk of otitis media by 5.20 times. This was confirmed in a study by Norhafizah et al. that examined the link between allergic rhinitis and otitis media and reported that the prevalence of allergic rhinitis in otitis media was 80%. Dust particles were the most common cause of allergies [18]. This finding has been confirmed in other studies [6,19].

The results showed that adenoid hypertrophy is one of the main causes of otitis media such that adenoid hypertrophy increases the risk of otitis media by 1.15 times. In a study on 2602 children, Kumari et al. identified adenoid hypertrophy as the most important risk factor for otitis media [4]. This was also found in a study by Amani and Khazraie [20].

The present study found a significant relationship between absolute breastfeeding and otitis media, which is consistent with a cohort study by Telee et al. in Boston [5]. Seasonal rhinitis was another risk factor for otitis media in the present study. This finding has also been proven in previous studies [6]. Another risk factor examined in the present study was working mothers, which was significantly different between the case and control groups and was one of the predictors of otitis media incidence. This finding has not been studied before. Rhinorrhea and tonsillopharyngitis risk factors were not significantly different between the two groups, which is similar to studies by Kumari et al. and Sophia et al. [4,6]. However, according to the logistic regression model, these factors were not predictors of otitis media in children.

This study found no link between family size and otitis media, but in the Norhafizah et al. study, the presence of more than four family members was a risk factor for otitis [18]. When comparing the two groups, there was no significant difference in other examined factors, including smoking, low birth weight, snoring, and vaccination. This may be due to the small sample size when examining the above factors. A study by Lubianca et al. also did not show a significant relationship between cigarette smoke and the incidence of otitis media [21]. Additionally Sophia et al. showed a link between snoring and the incidence of otitis media [6].

One of the limitations of this study was the small sample size and the time-consuming nature of examining all risk factors in all patients during the research period.

## Conclusions

Although pacifiers and bottle feeding, seasonal rhinitis, allergic rhinitis, adenoid hypertrophy, working mother, and rhinorrhea are among the risk factors for otitis media, allergic rhinitis and adenoid hypertrophy are two risk factors that are stronger predictors of otitis media. Therefore, pediatricians should increase their awareness of the existence of these risk factors in a patient and consider all risk factors in otitis media for appropriate diagnostic and therapeutic measures to prevent the complications of otitis media such as speech, language, and cognitive delay.
